# High-throughput proteomics profiling-derived signature associated with chemotherapy response and survival for stage II/III colorectal cancer

**DOI:** 10.1038/s41698-023-00400-0

**Published:** 2023-05-31

**Authors:** Shu-Biao Ye, Yi-Kan Cheng, Pei-Si Li, Lin Zhang, Lian-Hai Zhang, Yan Huang, Ping Chen, Yi Wang, Chao Wang, Jian-Hong Peng, Li-Shuo Shi, Li Ling, Xiao-Jian Wu, Jun Qin, Zi-Huan Yang, Ping Lan

**Affiliations:** 1 Guangdong Institute of Gastroenterology; Guangdong Provincial Key Laboratory of Colorectal and Pelvic Floor Diseases, The Sixth Affiliated Hospital, Sun Yat-sen University, Guangzhou, Guangdong PR China; 2 Department of Colorectal Surgery, Department of General Surgery, The Sixth Affiliated Hospital, Sun Yat-sen University, Guangzhou, Guangdong PR China; 3 Department of Radiation Oncology; The Sixth Affiliated Hospital, Sun Yat-sen University, Guangzhou, Guangdong PR China; 4 Sun Yat-sen University School of Medicine, Sun Yat-sen University, Guangzhou, PR China; 5 Department of Clinical Laboratory, Sun Yat-sen University Cancer Center; State Key Laboratory of Oncology in South China; Collaborative Innovation Center for Cancer Medicine, Guangzhou, PR China; 6 Department of Surgery, Peking University Cancer Hospital and Institute, Beijing, PR China; 7 Department of Pathology, The Sixth Affiliated Hospital, Sun Yat-sen University, Guangzhou, Guangdong PR China; 8 Department of VIP, Sun Yat-sen University Cancer Center; State Key Laboratory of Oncology in South China; Collaborative Innovation Center for Cancer Medicine, Guangzhou, PR China; 9 State Key Laboratory of Proteomics, National Center for Protein Sciences (The PHOENIX Center, Beijing), Beijing Proteome Research Center, Beijing, China; 10 Department of Colorectal Surgery, Sun Yat-sen University Cancer Center; State Key Laboratory of Oncology in South China; Collaborative Innovation Center for Cancer Medicine, Guangzhou, PR China; 11 Department of Probability and Statistics, The Sixth Affiliated Hospital, Sun Yat-sen University, Guangzhou, PR China; 12 Department of Probability and Statistics, School of Public Health, Sun Yat-sen University, Guangzhou, PR China

**Keywords:** Predictive markers, Prognostic markers, Colorectal cancer

## Abstract

Adjuvant chemotherapy (ACT) is usually used to reduce the risk of disease relapse and improve survival for stage II/III colorectal cancer (CRC). However, only a subset of patients could benefit from ACT. Thus, there is an urgent need to identify improved biomarkers to predict survival and stratify patients to refine the selection of ACT. We used high-throughput proteomics to analyze tumor and adjacent normal tissues of stage II/III CRC patients with /without relapse to identify potential markers for predicting prognosis and benefit from ACT. The machine learning approach was applied to identify relapse-specific markers. Then the artificial intelligence (AI)-assisted multiplex IHC was performed to validate the prognostic value of the relapse-specific markers and construct a proteomic-derived classifier for stage II/III CRC using 3 markers, including FHL3, GGA1, TGFBI. The proteomics profiling-derived signature for stage II/III CRC (PS) not only shows good accuracy to classify patients into high and low risk of relapse and mortality in all three cohorts, but also works independently of clinicopathologic features. ACT was associated with improved disease-free survival (DFS) and overall survival (OS) in stage II (pN0) patients with high PS and pN2 patients with high PS. This study demonstrated the clinical significance of proteomic features, which serve as a valuable source for potential biomarkers. The PS classifier provides prognostic value for identifying patients at high risk of relapse and mortality and optimizes individualized treatment strategy by detecting patients who may benefit from ACT for survival.

## Introduction

Colorectal cancer (CRC) remains a major public health problem and is the third most common cancer and the third leading cause of cancer-related death among men and women^1^. Of the 1,900,000 new cases of colorectal cancer annually, approximately 70% of CRC patients are diagnosed with stage II/III disease. To reduce the risk of cancer recurrence and improve survival, fluorouracil-based adjuvant chemotherapy (ACT) is recommended as the standard treatment for stage III CRC and some high-risk stage II CRC (e.g., T4, high grade, fewer than 12 lymph nodes examined) after surgery^2^. However, ACT may only provide additional survival benefit in certain subsets of patients. Currently, the selection of patients is suboptimal, which leads to either over- or undertreatment^3^. A previous study has reported that 50% of stage III patients are cured by surgery alone, and 20% of those can survival with the addition of ACT. Altogether, only 20% of stage III CRC patients really benefit from ACT, exposing 80% of those to unnecessary toxicity^4^. In stage II patients, the role of ACT remains an area of great controversy because only a subset of patients will yield considerable benefit. Even though the QUASAR clinical trial revealed that ACT could improve survival of patients with stage II CRC, the absolute improvements were small (approximately 3.6%)^5^. Furthermore, up to 30% of stage II CRC patients will experience relapse, which is generally fatal^6^. Therefore, the current staging system is not sufficient for management in patients with stage II/III CRC, and it is crucial to identify biomarkers for detecting patients who could benefit from ACT.

Mass spectrometry (MS)-based proteomic is a promising technique for the discovery of diagnostic and prognostic methods and the identification of prognostic signatures of proteins^7–9^, which are usually the final executors of biological activities. Thus, proteomic (proteomic-derived signature) has been successfully applied in improvement of diagnostic accuracy^10,11^, response to therapy^12,13^, and prognosis prediction^14^. Moreover, proteomic might, in theory, objectively reflect the tumor’s biology nature to relate patient’s prognosis. Specifically, proteomic has been showed to be an effective tool to predict prognosis^15^ and response to treatment^16^ in CRC. However, few studies have focused on the prediction of postoperative survival and ACT benefit.

In the present study, we investigate the comprehensive proteomic profiling to explore the clinical significance of proteomic features in stage II/III CRC, and then develop and validate a proteomic signature to predict disease-free survival (DFS) and overall survival (OS) in multicenter cohorts. With this proteomic signature and pathologic stage, we further detect the subset of patients that could benefit from ACT.

## Results

### Proteomic profiling of discovery cohort

**Fig. 1:**
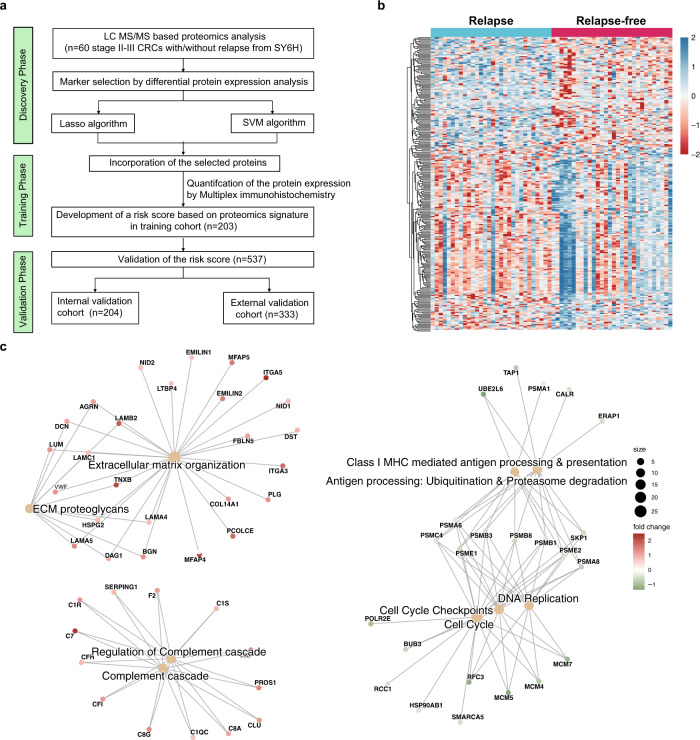
Outline of workflow and proteomic landscape of patients from discovery cohort. **a** Study design and flow chart. **b** Heatmap of proteins significantly associated with recurrence in CRC. **c** GSEA (H: Reactome) analysis of stage II/III CRC patients revealed the pathways associated with relapse (*n* = 30) or non-relapse (*n* = 30). LC-MS/MS Liquid chromatography tandem mass spectrometry, CRC Colorectal cancer, SY6H Sun Yat-sen University, the Sixth affiliated Hospital.

The workflow of present study and proteomic landscape is shown in Fig. 1 and Supplementary Fig. 1. The baseline clinical parameters were well balanced in 60 CRC patients with and without relapse in terms of sex, age, T stage, N stage, and treatment, which rules out the potential impact of these factors on relapse (Supplementary Table 2). QC samples (293 T cell) were routinely assayed as quality control samples to guarantee good reproducibility and sensitivity (Supplementary Fig. 2). We then explored detailed protein expression patterns by using mass spectrometry (MS)-based high-throughput assay between two pre-defined subject CRC groups in discovery cohort: relapse and relapse-free (Fig. 1b) and found distinct profiles. GSEA identified extracellular matrix organization (ECM), ECM proteoglycans, complement cascade upregulated in the relapse group, while antigen processing, presentation, and cell cycle downregulated compared to relapse-free group. (Fig. 1c).

### Clinicopathological characteristics of the training and validation cohorts

A total of 740 pretreatment, stage II/III CRC specimens obtained from patients at 3 academic institutions were included in our analysis. The baseline demographic and clinicopathological features of patients in the training cohort (*n* = 203), internal validation cohort (*n* = 204), and external validation cohort (*n* = 333) are shown in Table 1. The median follow-up time was 104.2 months (IQR 66.6−116.2) in the training cohort, 108.4 months (IQR 78.2−118.8) in the internal validation cohort, and 76.7 months (IQR 34.7−88.3) in the external validation cohort.

**Table 1:** Clinical characteristics of patients in the training, internal, and external validation cohorts.

	Training cohort (*n* = 203)			Internal validation cohort (*n* = 204)			External validation cohort (*n* = 333)		
	Patients	Low PS (*n* = 77)	High PS (*n* = 126)	Patients	Low PS (*n* = 63)	High PS (*n* = 141)	Patients	Low PS (*n* = 104)	High PS (*n* = 229)
Age (years)									
< 65	129 (63.5)	51 (66.2)	78 (61.9)	131 (64.2)	43 (68.3)	88 (62.4)	225 (67.6)	70 (67.3)	155 (67.7)
≥ 65	74 (36.5)	26 (33.8)	48 (38.1)	73 (35.8)	20 (31.7)	53 (37.6)	108 (32.4)	34 (32.7)	74 (32.3)
Sex									
Female	84 (41.4)	37 (48.1)	47 (37.3)	81 (39.7)	30 (47.6)	51 (36.2)	142 (42.6)	41 (39.4)	101 (44.1)
Male	119 (58.6)	40 (51.9)	79 (62.7)	123 (60.3)	33 (52.4)	90 (63.8)	191 (57.4)	63 (60.6)	128 (55.9)
Tumor location									
Colon	112 (55.2)	42 (54.5)	70 (55.6)	106 (52.0)	35 (55.6)	71 (50.4)	195 (58.6)	65 (62.5)	130 (56.8)
Rectum	91 (44.8)	35 (45.5)	56 (44.4)	98 (48.0)	28 (44.4)	70 (49.6)	138 (41.4)	39 (37.5)	99 (43.2)
Histology type									
High	158 (77.8)	61 (79.2)	97 (77.0)	162 (79.4)	54 (85.7)	108 (76.6)	260 (78.1)	89 (85.6)	171 (74.7)
Low	42 (20.7)	16 (20.8)	26 (20.6)	40 (19.6)	7 (11.1)	33 (23.4)	57 (17.1)	13 (12.5)	44 (19.2)
Missing	3 (1.5)	0 (0.0)	3 (2.4)	2 (1.0)	2 (3.2)	0 (0.0)	16 (4.8)	2 (1.9)	14 (6.1)
pT stage									
pT1	2 (1.0)	0 (0.0)	2 (1.6)	1 (0.5)	0 (0.0)	1 (0.7)	1 (0.3)	1 (1.0)	0 (0.0)
pT2	5 (2.5)	3 (3.9)	2 (1.6)	11 (5.4)	3 (4.8)	8 (5.7)	9 (2.7)	2 (1.9)	7 (3.1)
pT3	174 (85.7)	67 (87.0)	107 (84.9)	166 (81.4)	53 (84.1)	113 (80.1)	274 (82.3)	87 (83.7)	187 (81.7)
pT4	22 (10.8)	7 (9.1)	15 (11.9)	26 (12.7)	7 (11.1)	19 (13.5)	49 (14.7)	14 (13.5)	35 (15.3)
pN stage									
pN0	97 (47.8)	49 (63.6)	48 (38.1)	101 (49.5)	36 (57.1)	65 (46.1)	172 (51.7)	55 (52.9)	117 (51.1)
pN1	82 (40.4)	21 (27.3)	61 (48.4)	80 (39.2)	22 (34.9)	58 (41.1)	107 (32.1)	38 (36.5)	69 (30.1)
pN2	24 (11.8)	7 (9.1)	17 (13.5)	23 (11.3)	5 (7.9)	18 (12.8)	54 (16.2)	11 (10.6)	43 (18.8)
Stage									
II	97 (47.8)	49 (63.6)	48 (38.1)	101 (49.5)	36 (57.1)	65 (46.1)	172 (51.7)	55 (52.9)	117 (51.1)
III	106 (52.2)	28 (36.4)	78 (61.9)	103 (50.5)	27 (42.9)	76 (53.9)	161 (48.3)	49 (47.1)	112 (48.9)
Number of lymph nodes examined									
< 12	15 (7.4)	6 (7.8)	9 (7.1)	11 (5.4)	2 (3.2)	9 (6.4)	117 (35.1)	35 (33.7)	82 (35.8)
≥ 12	188 (92.6)	71 (92.2)	117 (92.9)	193 (94.6)	61 (96.8)	132 (93.6)	216 (64.9)	69 (66.3)	147 (64.2)
CEA concentration									
< 5	136 (67.0)	53 (68.8)	83 (65.9)	122 (59.8)	42 (66.7)	80 (56.7)	193 (58.0)	61 (58.7)	132 (57.6)
≥ 5	57 (28.1)	18 (23.4)	39 (31.0)	71 (34.8)	18 (28.6)	53 (37.6)	130 (39.0)	36 (34.6)	94 (41.0)
Missing	10 (4.9)	6 (7.8)	4 (3.2)	11 (5.4)	3 (4.8)	8 (5.7)	10 (3.0)	7 (6.7)	3 (1.3)
Adjuvant chemotherapy									
Yes	109 (53.7)	49 (63.6)	60 (47.6)	104 (51.0)	30 (47.6)	74 (52.5)	227 (68.2)	74 (71.2)	153 (66.8)
No	63 (31.0)	20 (26.0)	43 (34.1)	74 (36.3)	23 (36.5)	51 (36.2)	75 (22.5)	20 (19.2)	55 (24.0)
Missing	31 (15.3)	8 (10.4)	23 (18.3)	26 (12.7)	10 (15.9)	16 (11.3)	31 (9.3)	10 (9.6)	21 (9.2)
Follow-up, years	8.68 (5.55−9.68)	9.05 (7.98−9.69)	8.55 (4.20−9.66)	9.03 (6.52−9.9)	9.22 (8.05−10.37)	8.74 (6.03−9.69)	6.39 (2.89−7.36)	6.48 (3.02−7.46)	6.06 (2.69−7.37)

Data are median (IQR) or *n* (%). *CEA* Carcinoembryonicantigen, *PS* Proteomic signature.

### Construction of proteomic signature

Twelve relevant proteins were identified by the coarse-to-fine feature selection strategy from discovery cohort. The least absolute shrinkage and selection operator (LASSO)/ SVM logistic model was applied into further selection and multiple immunohistochemistry was used to build the proteomic signature including FHL3, GGA1, TGFBI (Supplementary Figs. 2, 3; Supplementary Tables 3, 4). The risk score of each patient was calculated using the following formula based on their regression coefficient of the expression levels of these 3 markers (Supplementary Table 4): risk score = 0.003 × Hscore of FHL3 in tumor -0.006× Hscore of GGA1 in tumor +0.004× Hscore of TGFBI in stromal. For each of the training cohort and the two validation cohorts, X-tile plots were used to generate an optimum cutoff value (Supplementary Fig. 7) to stratify patients into high- and low-proteomic signature groups for further analyses.

### Association between proteomic signature and prognosis

**Fig. 2:**
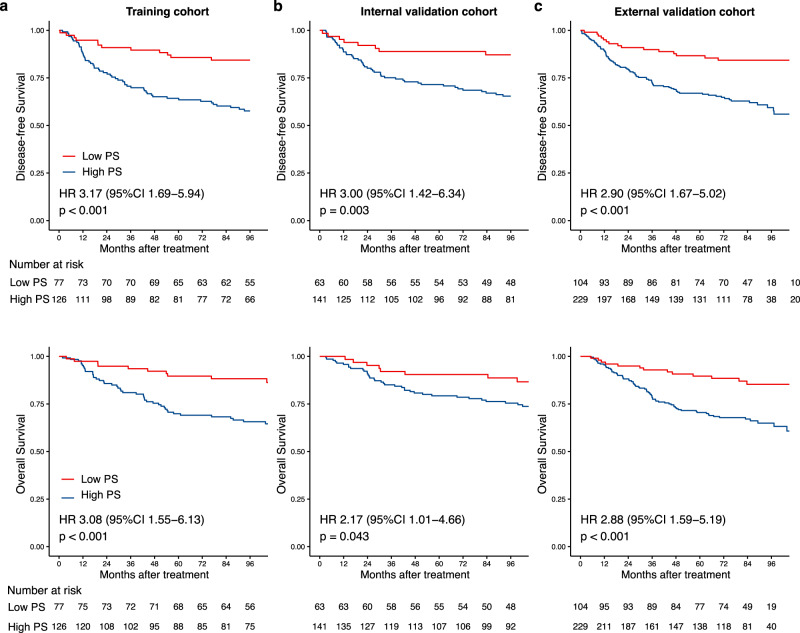
Kaplan-Meier curves for DFS and OS according to the PS. **a** Training cohort (upper: DFS, lower: OS, *n* = 203), (**b**) internal validation cohort (upper: DFS, lower: OS, *n* = 204), and (**c**) external validation cohort (upper: DFS, lower: OS, *n* = 333). We calculated the p values using the unadjusted log-rank test and hazard ratios using a univariate Cox regression analysis. DFS Disease-free survival, OS Overall survival, PS Proteomic signature, HR Hazard ratio, CI Confidential interval.

**Fig. 3:**
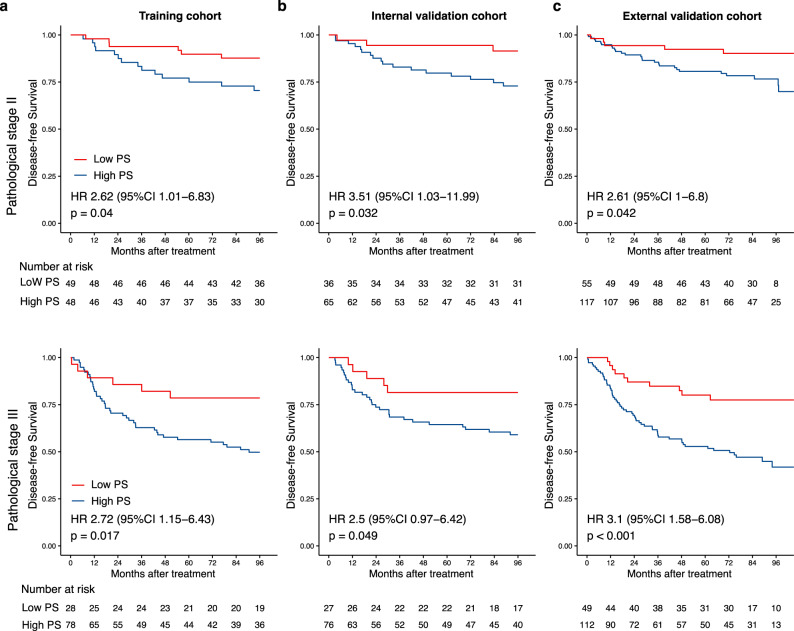
Kaplan-Meier curves for DFS according to the PS among stage II/III CRC patient subgroups. **a** The training cohort (upper: stage II, *n* = 97; lower: stage III, *n* = 106), (**b**) internal validation cohort (upper: stage II, *n* = 101; lower: stage III, *n* = 103), (**c**) external validation cohort (upper: stage II, *n* = 172; lower: stage III, *n* = 161). We calculated the *p*-values using the unadjusted log-rank test and hazard ratios using a univariate Cox regression analysis. DFS Disease-free survival, PS Proteomic signature, CRC Colorectal cancer, HR Hazard ratio, CI Confidential interval.

In all three cohorts, the Kaplan–Meier survival curves have revealed a significant difference in DFS between the high and low- proteomic signature groups (*p* < 0.005), with relatively high hazard ratios (HRs, > 2.9) (Fig. 2a–c, upper). Furthermore, a significant difference in OS was also confirmed between the high- and low- proteomic signature groups (*p* < 0.05), with hazard ratios (HRs, > 2.1) (Fig. 2a–c, lower). The number of patients who had an event for each risk group among each cohort and DFS, and OS outcomes are listed in the appendix (Supplementary Tables 5, 6). Subgroup analyses further revealed that the proteomic signature was a predictor for DFS stratified by clinical stage (Fig. 3) from each cohort.

**Fig. 4:**
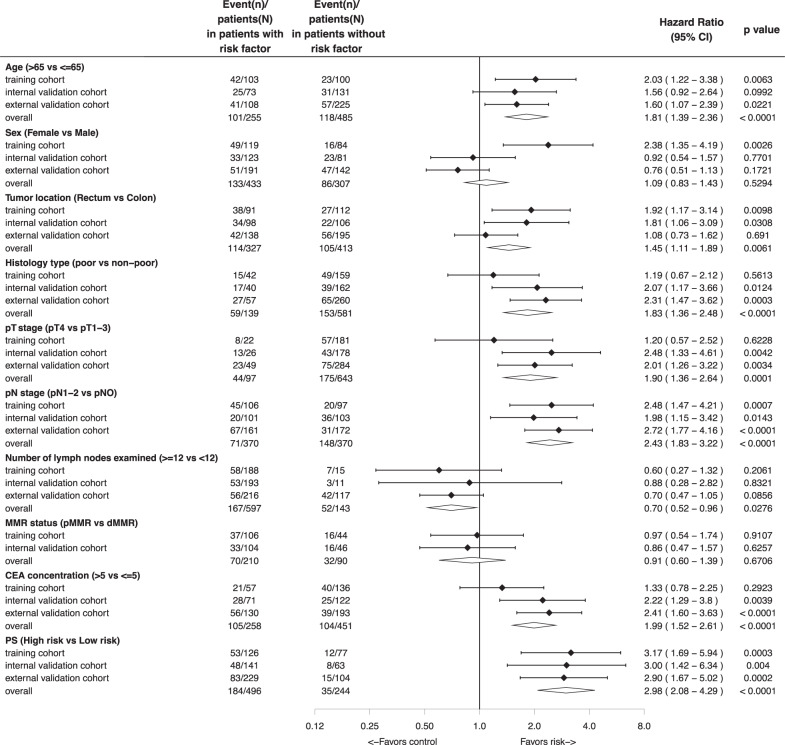
Univariate analysis of the PS and clinicopathological characteristics with DFS. PS Proteomic signature, DFS Disease-free survival.

The results of the univariate analysis of DFS by clinicopathological and proteomic signature subgroups in the three cohorts are shown in Fig. 4. After adjusting for the clinicopathological variables and the CEA level, multivariate analysis showed that proteomic signature was associated with DFS in the training cohort (HR 2.62, 95% CI 1.38−4.96, *p* = 0.003, Table 2), as well as in the internal validation cohort (HR 2.81, 95% CI 1.33−5.96, *p* = 0.007) and the external validation cohort (HR 2.84, 95% CI 1.61−5.02, *p* < 0.001). Moreover, proteomic signature was associated with OS in the training cohort (HR 2.53, 95% CI 1.26−5.10, *p* = 0.009, Supplementary Table 7) and the external validation cohort (HR 2.93, 95% CI 1.58−5.42, *p* < 0.001). These survival results demonstrated the high prognostic accuracy of the proteomic signature.

**Table 2:** Multivariable DFS analyses in each cohort.

Variables	HR	95% CI	*P*-value
Training cohort (*n* = 203)			
Age (≥ 65 vs. < 65)	2.02	1.21−3.63	0.007
Tumor location (rectum vs. colon)	1.96	1.19−3.22	0.008
pN stage (N1-2 vs. N0)	1.97	1.15−3.37	0.014
PS (high vs. low)	2.62	1.38−4.96	0.003
Internal validation cohort (*n* = 204)			
pT stage (T4 vs. T1-3)	2.75	1.46−5.17	0.002
pN stage (N1-2 vs. N0)	2.01	1.15−3.52	0.014
PS (high vs. low)	2.81	1.33−5.96	0.007
External validation cohort (*n* = 333)			
pT stage (T4 vs. T1-3)	2.29	1.40−3.75	< 0.001
N stage (N2 vs. N1)	2.95	1.90−4.56	< 0.001
CEA ( ≥ 5 vs. <5 ng/L)	2.28	1.51−3.43	< 0.001
PS (high vs. low)	2.84	1.61−5.02	< 0.001

We calculated hazard ratios and p values using an adjusted multivariate Cox proportional hazards regression model, including proteomic signature (high risk vs. low risk), sex (male vs. female), age (≥ 60 years vs. 60 years), pN stage (N1-2 vs. N0), pT stage (T4 vs. T1-3), MMR, (dMMR vs. pMMR) histology (low vs. high), location (rectum vs. colon), lymph nodes examined (≥ 12 vs. < 12). and CEA ( ≥ 5 vs. < 5 ng/L). *DFS* Disease-free survival, *PS* Proteomic signature, *CEA* Carcinoembryonic antigen, *HR* Hazard ratio, *CI* Confidence interval. We selected variables with the backward stepwise approach, the p value threshold was 0·05 (*p* > 0·05) for removing insignificant variables from the model. Only variables that were significantly associated with survival are presented.

### Prognostic accuracy of proteomic signature integrated with clinicopathologic features

**Fig. 5:**
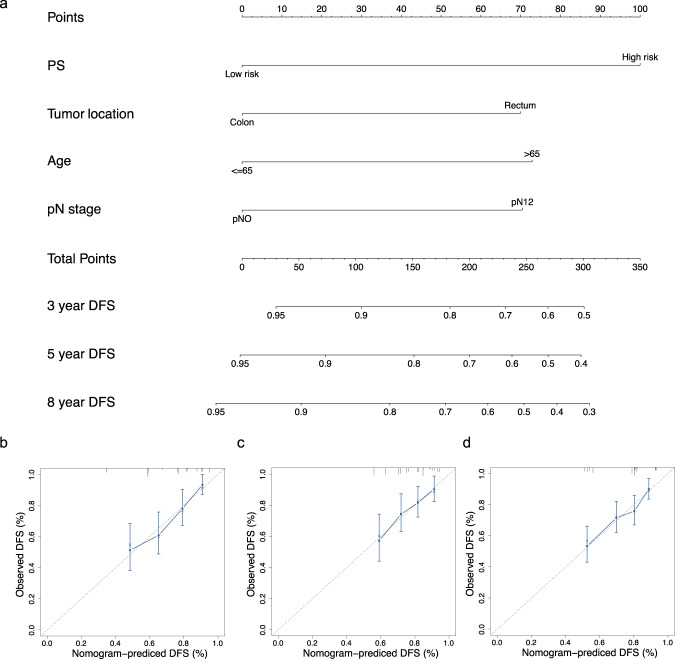
Nomogram to predict the risk of recurrence in stage II/III CRC patients. **a** Nomogram to predict DFS. Calibration curves to predict 8-year disease-free survival in (**b**) the training cohort, (**c**) the internal validation cohort, and (**d**) the external validation cohort; The nomogram-predicted probability is plotted on the x-axis and the actual survival is plotted on the y-axis. PS Proteomic signature, CRC Colorectal cancer, ROC Receiver operator characteristic, DFS Disease-free survival.

In addition, multivariable analysis was performed to generate a nomogram to predict 8-year DFS in the training cohort using the predictors including age, tumor location, N stage, and proteomic signature (Fig. 5a, Supplementary Table 8). Among these predictors, the proteomic signature had the highest C-index. The calibration plots for the nomogram of the 8-year DFS were predicted well in the training cohort (C-index 0.78, 95% CI 0.71–0.85), the internal validation cohort (0.78, 0.72–0.84), and the external validation cohort (0.75, 0.68–0.82; Fig. 5b–d, Supplementary Fig. 8). The ability of the proteomic signature to predict DFS was superior to that of existing risk factors such as N stage, primary tumor location, and age (Supplementary Fig. S8).

### Association between proteomic signature and benefit from ACT

**Fig. 6:**
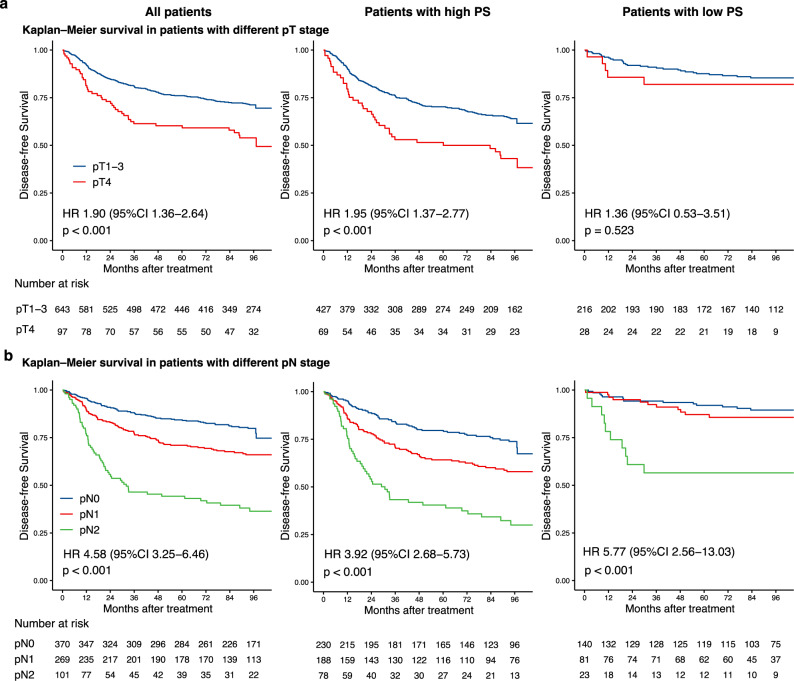
Kaplan-Meier curves for DFS according to the PS. The results are shown for all patients (*n* = 740, left), patients with a high PS (*n* = 496, middle), and patients with a low PS (*n* = 244, right). The results are also stratified according to pT stage (**a**), and pN stage (**b**). *p*-values were calculated using two-sided log-rank test. PS Poteomic signature, DFS Disease-free survival, HR Hazard ratio, CI Confidential interval.

**Fig. 7:**
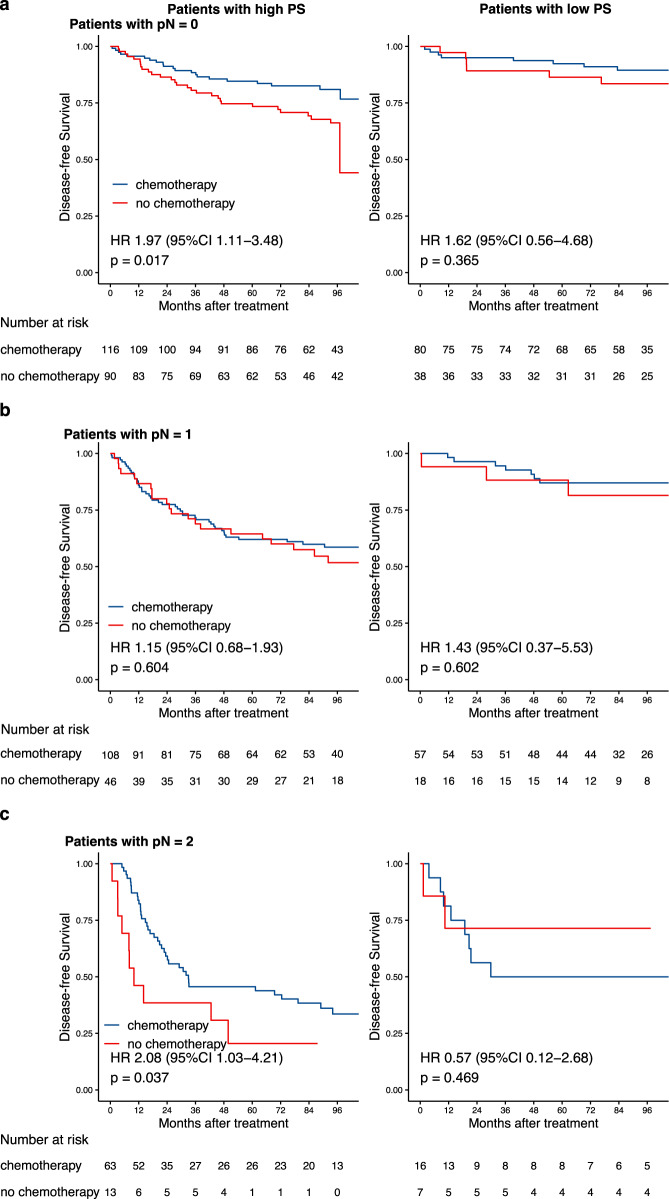
ACT benefits based on DFS according to pN stage and PS. **a**–**c** Kaplan-Meier DFS curves are shown for patients according to their use of ACT. In addition, patients with a high PS (left) were stratified according to pN0 (*n* = 206, upper), pN1 (*n* = 154, middle), and pN2 (*n* = 76, bottom). Patients with a low PS (right) were also stratified according to pN0 (*n* = 118, upper), pN1 (*n* = 75, middle), and pN2 (*n* = 23, bottom). p values were calculated using two-sided log-rank test. PS Proteomic signature, DFS Disease-free survival, HR Hazard ratio, CI Confidential interval.

In order to verify the clinical significance of proteomic signature for detecting patients that could benefit from ACT, subgroup analyses stratified by proteomic signature, pathological stage and ACT were performed. Subgroup analyses indicated that both pT stage and pN stage were correlated with DFS (Fig. 6a, b) and OS (Supplementary Fig. 9) among all patients. In the high-proteomic signature group, both pT and pN stage were significantly associated with DFS (HR: 1.90, 95% CI: 1.36–2.64, *p* < 0.001 in pT stage, and *p* < 0.001 in pN stage, Fig. 6) and OS (HR: 2.07, 95% CI: 1.45–2.94, *p* < 0.001 in pT stage, and *p* < 0.001 in pN stage, Supplementary Fig. 9). In the low proteomic signature group, only pN stage was significantly associated with DFS (*p* < 0.001) and OS (*p* < 0.001). Subgroup analysis for pN stage with high-proteomic signature revealed that, stage II (pN0) patients with ACT, had better DFS (HR: 1.97, 95% CI: 1.11–3.48, *p* = 0.017) (Fig. 7a) and OS (HR: 3.03, 95% CI: 1.49–6.17, *p* = 0.001) (Supplementary Fig. 10) than those without ACT, and pN2 patients had survival benefit from the ACT (HR: 2.08, 95% CI: 1.03–4.21, *p* = 0.037) for DFS (Fig. 7c) and OS (HR: 2.65, 95% CI: 1.28–5.47, *p* = 0.006) (Supplementary Fig. 10). Subgroup analyses indicate that not all stage II/III CRC patients will benefit from ACT, and not only pathological stage, but also proteomic signature could serve as a powerful tool to optimize decision making regarding ACT treatment strategy.

## Discussion

This study not only developed and validated a robust proteomic signature from comprehensive proteomic profiling associated with tumor relapse and survival of stage II/III CRC patients, but also investigated the association between the proteomic signature and ACT efficacy. We revealed heterogeneity of CRC with and without relapse in proteomic features. More importantly, the present study could identify patients who can benefit from ACT through the stratification of the proteomic signature and pathological stage.

CRC patients with the same stage who receive similar treatment might have different clinical outcomes, which makes accurate prognostication essential for treatment planning. Previous studies have indicated the prognostic value or drug sensitivity of proteomic features in CRC^7,17,18^ and few proteomic biomarkers have been applied in clinical practice due to the small sample sizes and a lack of large-scale validation cohorts. Our recent study applied proteomic analysis to define proteomic signature for progression of gastric lesion and validate their value via IHC^11^. Similarly, the present multicenter study revealed a proteomic signature predicting survival and ACT efficacy in stage II/III colorectal cancer.

The most important finding of this study was that this classifier could serve as a powerful tool for optimizing decision-making on ACT for stage II/III CRC. Stratified with proteomic signature and pathological stage, for patients with stage II disease in the high-proteomic signature group, receiving ACT may indicate better prognosis compared with not receiving ACT, and patients with pN2 disease in the high-proteomic signature group experienced a substantial benefit from ACT. Although current guidelines recommend ACT for most stage II/III CRC patients^19,20^, some studies have demonstrated that not all patients will benefit from ACT^3,4^. Our findings are consistent with previous reports that patients with stage II disease and a high-risk feature have to receive ACT and more aggressive systemic therapy should be considered for patients with pN2 disease and a poor prognosis. Thus, the proteomic signature might provide a new stratification method for identifying patients who should and should not receive ACT.

We validated proteomic signature including three proteins- GGA1, FHL3, TGFBI, associated with disease progression and efficacy of chemotherapy. Of them, transforming growth factor-beta-induced protein (TGFBI), as an extracellular matrix (ECM) protein, has indicated a critical role in tumor progression, angiogenesis^21^, and sensitivity of 5-Fluorouacil based chemotherapy in CRC^22,23^. TGFBI is frequently methylated and associated with chemotherapy resistance^24^. Much evidence has demonstrated that TGFBI is secreted by macrophages and had a role in immunosuppression in cancers^25,26^. Andrei Turtoi. et al. employed proteomics analysis and identified proteomic signature including TGFBI was associated with CRC liver metastasis^27^. The above studies provided evidence that TGFBI might affect tumor prognosis, chemotherapy efficacy and may be an effector of the tumor-promoting actions of TGFβ and a potential therapeutic target. Another protein has also indicated potential importance previously. Four and a half LIM domains 3 (FHL3), as a member of FHL proteins, was identified to be a novel TGF-beta-like signaling pathway and indicates a useful molecular target for cancer therapy^28^. Several studies implied that FHL3 contributed to tumor metastasis^29^ and EMT, and chemotherapy resistance^30^. The expression patterns of proteins in the signature may provide new insights into the molecular mechanisms that underlie tumor relapse and chemotherapy resistance, thus could provide potential novel targets and treatment strategies for CRC patients.

Using proteomic analysis and IHC as our recent study reported^11^, we identified and validated a proteomic signature to predict prognosis and ACT efficacy. Tissue is very commonly applied for biomarker detection in operative samples or biopsies. For example, the detection of MMR proteins by IHC is currently recommended for deciding the application of immunotherapy in metastatic CRC according to the guideline^19^. The application of our surgical tissue proteomic signatures may potentially provide information about postoperative prognosis and ACT efficacy for stage II/III CRC patients, thus help them decide for further appropriate management strategies.

The present study has several limitations that merit consideration. The first is the retrospective data collection and limited sample size. Although this study was performed following the REMARK guidelines^31^ and consecutive patients were enrolled from multicenter cohorts, the signature has not yet been validated in prospective studies; we are currently performing a prospective study to validate our findings (NCT03025854). Second, the biological functions of these molecules in carcinogenesis and development needs to be further explored, even though previous studies indicated their importance in cancer development and chemotherapy efficacy. Third, the performance of the proteomic signature was only examined in Chinese patients, and future studies are warranted to validate its performance in different ethnic populations.

In conclusion, we developed a proteomic signature that effectively predicted prognosis in stage II/III CRC patients. The prognostic value of classifier was validated in independent populations. Combination of the proteomic signature with pathological stage might provide an aid in selecting which patients might benefit from ACT. Larger-scale, prospective studies are warranted before regulatory approval of clinical routine application of key protein signatures.

## Methods

### Patient cohorts and tumor specimens

This study complied with the REMARK guidelines for tumor marker prognostic studies (Supplementary Table 1). In the discovery phase, proteomic profiling analysis was conducted on tumor samples and adjacent normal colorectal mucosa from 60 patients with stage II/III CRC (Supplementary Table 2). In the training and validation phase, we analyzed a cohort of patients with stage II-III colorectal cancer who received treatment at 3 academic centers in China. The patients in training and internal validation cohorts originated from the institutional database program of colorectal disease (IDPCD) at the Sixth Affiliated Hospital, Sun Yat-sen University^32^, which has prospectively enrolled CRC patients and integrated the patients from our National Key Research and Development Project of CRC Screen, Surveillance, and Intervention^33^. The patients in external validation cohort originated from tumor registry at 2 academic cancer centers. We excluded samples if the patient met the exclusion criteria (clinical quality control, eg, metastatic cancer, received previous treatment with any anticancer therapy, stage I disease, or missing mortality or recurrence data). All patients received curative-intent surgery, and no patients received preoperative antitumor treatment. After radical surgery, a proportion of patients received available standard systemic treatment, include fluorouracil (FU) or capecitabine with or without oxaliplatin. We included 740 samples which passed quality control for the final analysis. The workflow for the development and validation of PS classifier have been detailed in Fig. 1a. This multicenter study was conducted in accordance with the Declaration of Helsinki. This study was approved by the Institutional Review Board of The Sixth Affiliated Hospital, Sun Yat-sen University (2020ZSLYEC-229), and written informed consent was obtained from all patients before treatment.

### Proteomic analysis

Tissue samples were prepared as previously described in ref. ^34^. In brief, tissues were lysed using 8 M urea lysis buffer followed by sonication. The protein was then reduced and alkylated using the FASP method. The digested peptides were separated into three fractions using a reverse-phase C18 column and a stepwise gradient of increasing acetonitrile concentration at pH 10. The experimental workflow of proteomic analysis in the discovery phase was shown in Supplementary Fig. 1a. Protein profiles were acquired on an Orbitrap Fusion and Orbitrap Fusion Lumos mass spectrometer (Thermo Fisher Scientific, Rockford, IL, USA) or a Q Exactive HF mass spectrometer (Thermo Fisher Scientific, Rockford, IL, USA)^34^. A data-dependent mode was performed by measuring MS1 in the Orbitrap at a resolution of 120,000 followed by up to 20 data-dependent MS/MS scans with higher-energy collision dissociation (normalized collision energy of 35%). Digested 293 T cells used as quality control samples were assayed daily to guarantee the sensitivity and reproducibility (Supplementary Figure 2). Raw files generated by MS experiments were submitted to Firmiana, a one-stop proteomic data processing platform^35^. Peptides with a false discovery rate (FDR) lee than 1% were selected and only proteins with high quality and unique peptides were considered qualified to minimize the FDR at protein level. We used label-free intensity-based absolute quantification (iBAQ) to quantify proteins^36^. The iBAQ values were then converted to the intensity-based fraction of total (iFOT) to perform further on data analysis^37^.

### Quantitative RT-PCR (qRT-PCR)

A FastPure Cell/Tissue Total RNA Isolation Kit V2 (Vazyme, Nanjing, China) was used to extract total RNA from cells and frozen specimens. Complementary DNA (cDNA) was synthesized by using the HiScript III RT SuperMix for qPCR (+gDNA wiper) (Vazyme, Nanjing, China). Following were the primer sequences used for RT-qPCR: GGA1, forwards TCACGGAGATGGTGATGAGCCA and reverse TCCTCTG TGTCACTCGCCAGTC; TGFBI, forwards GGACATGCTCACTATCAACGGG and reverse CTGTGGACACATCAGACTCTGC; FHL3, forwards ACAAGGGTGCTCAC TACTGCGT and reverse TTCTCGATGCCACGGCTGATCA; NDUFS7, forwards AGGCACGAGGTGTCCATCAGAG and reverse CAGTTGACGAGGTCATCCAGC T; glyceraldehyde 3-phosphate dehydrogenase (GAPDH), forward CCAAAATCAGAT GGGGCAATGCTGG and reverse TGATGGCATGGACTGTGGTCATTCA.

### Immunofluorescence (IF)

Cells were previously seeded onto glass coverslips overnight, fixed with 4% paraformaldehyde for 15 min, and then penetrated with 0.5% Triton X-100 for 30 min at room temperature. After washing with PBS for three times, the cells were incubated with primary antibodies (1:100) against target proteins in blocking buffer at 4 °C overnight and with the corresponding secondary antibodies for 1 h at room temperature. Then, the ProLongTM Glass Antifade Mountant with NucBlueTM (Invitrogen, USA) was applied to mount the fixed cells for 5 min at room temperature, and the fixed cells were kept in the dark at 4 °C. Microscopy detection was performed, and images were analyzed under a Zeiss Axioskop-2 microscope.

### Vector’s construction and Transfection

The cDNA of GGA1, NDUFSF7, TGFB1 and FHL3 were amplified from HCT116 cell line and respectively cloned into pCDH-CMV-MCS-EF1-Puro vector. The Lipofectamine™ 3000 Reagent (Invitrogen) was used to mediate the plasmid containing the target gene into cells according to the recommendation of protocol. The transient transfection of plasmids and siRNAs were performed using the Lipofectamine 3000 kit (Invitrogen, USA) according to the recommendation of protocol. The siRNA sequences for transfection are listed as follow: TGFBI: CCACTACATTGATGAGCTA; FHL3: TCGAGAATGTCTGGTCTGT; NDUFS7: GGCACACTCACCAACAAGA; GGA1: GGTCGTGTCTCCCAAGTAT.

### Detection of mismatch repair (MMR)

Immunohistochemistry (IHC) staining was performed to detect the MMR status in primary tumor specimens by using antibodies targeting MLH1 (clone ES05; Zhong Shan Jin Qiao, Beijing, China, 1:40), MSH2 (clone RED2; Zhong Shan Jin Qiao, Beijing, China, 1:200), MSH6 (clone UMAB258; Zhong Shan Jin Qiao, Beijing, China, 1:200) and PMS2 (clone EP51; Zhong Shan Jin Qiao, Beijing, China, 1:40). Tumors showing the loss of at least one MMR protein by IHC in any tumor nuclei were designated as MMR deficient (dMMR), whereas those tumors with intact expression in all tumor nuclei were designated as MMR proficient (pMMR). The positive nuclear staining of lymphocytes, stromal cells and normal epithelial cells served as internal controls.

### Multiple immunohistochemistry (mIHC)

Then artificial intelligence (AI)-assisted multiplex IHC (Supplementary Fig. 1b) was performed to develop and validate the prognosis value of the relapse-specific markers. After the specificities of the antibodies employed were validated by siRNA knockdown or recombinant expression via IF (Fig. S3, S4). A multiplex IHC platform was constructed, and the stability of the platform was verified with a variety of antibodies. Validated primary antibodies including GGA1 (H00026088-M01, NOVUS, USA, 1:200), FHL3 (11028-2-AP, Proteintech, China, 1:300), NDUFS7 (15728-1-AP, Proteintech, China, 1:100) and TGFBI (ab170874, Abcam, USA, 1:400) were sequentially applied, followed by horseradish peroxidase (HRP)-conjugated secondary antibody incubation and tyramide signal amplification (Supplementary Fig. 4, Supplementary Table 3). In AI-assisted analyses, identification of the tumor region and intratumoral stromal region was performed through inForm following the steps check-train-confirm. inForm software was used to determine Hscore of each marker in tumor area or intratumoral stromal area. The data were normalized for further analysis. Then, we found that 3 of 4 proteins were mainly expressed in tumor cells, while TGFBI was more highly expressed in stromal cells than in tumor cells (Supplementary Figs. 3, 4).

### PS classifier construction

In the discovery stage, differentially expressed proteins (DEP) were identified as we previously described^11^. Wilcoxon test was used to perform the DEP analysis between the tissue groups to identify relapse-specific DEPs (relapse vs non-relapse tumors) and cancer-specific DEPs (tumor vs normal tissues). The least absolute shrinkage and selection operator (LASSO)/ SVM logistic-based machine learning approach^38,39^ was applied into further selection and was used to build the PS including the specific proteins (Supplementary Figure 5-6**;** Supplementary Table 4). The risk score of each patient was calculated using the following formula based on their regression coefficient of the expression levels of these markers: Risk score = β_1_χ_1_ + β_2_χ_2_ + β_3_χ_3_ +…… + β_n_χ_n_. The regression coefficient was calculated by the COX model. For each of the training cohort and the two validation cohorts, X-tile plots were used to generate an optimum cutoff value (Supplementary Fig. 7) to stratify patients into high- and low-PS groups for further analysis.

### Bioinformatics and statistical analysis

The primary endpoint was DFS, defined as the duration from surgery to the first observation of disease relapse (local or distant disease) or death from any cause. An additional endpoint was overall survival (OS) defined as the duration from surgery to death due to any cause. Kaplan-Meier methods were used to assess the association between the variables and survival, and the log-rank test was used to compare survival curves. Hazard ratios (HRs) were calculated by Cox regression analysis. In order to detect the subset of patients that could benefit from ACT, stratified analyses were performed according to the pathologic stage and PS associated with chemotherapy efficacy. The area under the curve (AUC) was calculated to evaluate the sensitivity and specificity of the model for predicting recurrence.

All statistical analyses were performed by R software (R Foundation for Statistical Computing, Vienna, Austria). Least Absolute Shrinkage and Selection Operator (LASSO) and support vector machine-recursive feature elimination (SVM-RFE) analyses were done using the “glmnet” package and e1071. Nomograms and calibration plots were generated using rms package. GSEA analysis was generated using “clusterprofiler” package^40^. P values less than 0.05 were considered statistically significant, and all statistical tests were two sided.

### Reporting summary

Further information on research design is available in the Nature Research Reporting Summary linked to this article.

## Supplementary information

SUPPLEMENTAL MATERIAL

REPORTING SUMMARY

## Data Availability

Deidentified clinical data can be made available with publication through the corresponding author after approval of a proposal with a signed data access agreement. Only deidentified data that underlie results reported in this Article can be shared with investigators who submit an approved proposal. After approval of a proposal, clinical data can be shared through a secure online platform after signing a data access agreement. The proteomic data has been deposited on iProX^41^ (https://www.proteomexchange.org/, accession number:IPX0003266000). iProX is an official member of ProteomeXchange Consortium^42^ which includes PRIDE^43^, PeptideAtlas^44^, MassIVE^45^, jPOST^46^, iProx, and Panorama Public^47^. The Consortium was established to provide globally coordinated standard data submission and dissemination pipelines involving the main proteomics repositories, and to encourage open data policies in the field.

## Code availability

No previously unreported custom computer codes or algorithms were used to generate or process the data presented in this manuscript.
