# Supplementary material for: High-throughput proteomics profiling-derived signature associated with chemotherapy response and survival for stage II/III colorectal cancer
Source: NPJ Precis Oncol. 2023 May 31;7:50. doi: 10.1038/s41698-023-00400-0 (PMC10232411; doi:10.1038/s41698-023-00400-0)
Supplement: Supplementary file 1 — SUPPLEMENTAL MATERIAL [file 41698_2023_400_MOESM1_ESM.docx]

**Supplementary Information**

**High-throughput proteomics profiling-derived signature associated with chemotherapy response and survival for stage II/III colorectal cancer**

**Table of Contents**

Search criteria used for “Evidence before this study”page 2

Expanded material and methods page 3

Supplementary Table 1 - REMARK checklist page 6

Supplementary Table 2 - Clinicopathological features of the 60 patients page 7

Supplementary Table 3 - Information of antibodies for four proteins page 8

Supplementary Table 4 - The association of DFS with 4 markers page 9

Supplementary Table 5 - Number of events page 10

Supplementary Table 6 - Five-year survival rates page 11

Supplementary Table 7 - Multivariable OS analysis page 12

Supplementary Table 8 - The C index, each cohort page 13

Supplementary Figure 1 - Experimental workflow page 14

Supplementary Figure 2 - Quality control of mass spectrometry page 15

Supplementary Figure 3 - Antibody validation for four proteins page 16

Supplementary Figure 4 - Multiplex IHC of three proteins value page 17

Supplementary Figure 5 – Marker selection page 18

Supplementary Figure 6 - Four proteins associated with DFS for ACT page 19

Supplementary Figure 7 - Determination of the optimum cutoff page 20

Supplementary Figure 8- ROC curves evaluate page 21

Supplementary Figure 9- Kaplan-Meier analysis for OS according to the PS page 22

Supplementary Figure 10- ACT benefits based on OS according to pN stage and PS page 23

1. **Search criteria used in “Evidence before this study”**

In the “Research in context” panel, the PubMed search outlined under “Evidence before this study” was the user query: ("proteomic") AND (prediction OR prognosis OR classification) AND (survival OR outcome) AND (colorectal cancer). PubMed translated this user query into the following detailed search query: ((proteomics [MeSH Terms]) OR (proteomics)) AND ((cancer, colorectal [MeSH Terms]) OR (colorectal cancer))AND (prediction [All Fields] OR ("prognosis" [MeSH Terms] OR "prognosis" [All Fields]) OR ("classification" [Subheading] OR "classification" [All Fields] OR "classification" [MeSH Terms])) AND (("mortality" [Subheading] OR "mortality" [All Fields] OR "survival" [All Fields] OR "survival" [MeSH Terms]) OR outcome [All Fields])

**Supplementary Table 1: REporting recommendations for tumor MARKer prognostic studies (REMARK) checklist**

| **No item** | **Item details** | **Where reported** | **Comments** |
| --- | --- | --- | --- |
| **INTRODUCTION** | | | |
| 1 | State the marker examined, the study objectives, and any pre-specified hypotheses. | Introduction, Methods, appendix |  |
| **MATERIALS AND METHODS** | | | |
| *Patients* | | | |
| 2 | Describe the characteristics (e.g., disease stage or co-morbidities) of the study patients, including their source and inclusion and exclusion criteria. | Methods |  |
| 3 | Describe treatments received and how chosen (e.g., randomized or rule-based). | Methods |  |
| *Specimen characteristics* | | | |
| 4 | Describe type of biological material used (including control samples) and methods of preservation and storage. | Methods, appendix |  |
| *Assay methods* | | | |
| 5 | Specify the assay method used and provide (or reference) a detailed protocol, including specific reagents or kits used, quality control procedures, reproducibility assessments, quantitation methods, and scoring and reporting protocols. Specify whether and how assays were performed blinded to the study endpoint. | Methods, appendix |  |
| *Study design* | | | |
| 6 | State the method of case selection, including whether prospective or retrospective and whether stratification or matching (e.g., by stage of disease or age) was used. Specify the time period from which cases were taken, the end of the follow-up period, and the median follow-up time. | Methods |  |
| 7 | Precisely define all clinical endpoints examined. | Methods |  |
| 8 | List all candidate variables initially examined or considered for inclusion in models. | Methods |  |
| 9 | Give rationale for sample size; if the study was designed to detect a specified effect size, give the target power and effect size. |  | Include as many as samples as possible to represent variation. |
| *Statistical analysis methods* | | | |
| 10 | Specify all statistical methods, including details of any variable selection procedures and other model-building issues, how model assumptions were verified, and how missing data were handled. | Methods, appendix |  |
| 11 | Clarify how marker values were handled in the analyses; if relevant, describe methods used for cutpoint determination. | Methods, appendix |  |
| **RESULTS** | | | |
| *Data* | | | |
| 12 | Describe the flow of patients through the study, including the number of patients included in each stage of the analysis (a diagram may be helpful) and reasons for dropout. Specifically, both overall and for each subgroup extensively examined report the numbers of patients and the number of events. | Results, appendix |  |
| 13 | Report distributions of basic demographic characteristics (at least age and sex), standard (disease-specific) prognostic variables, and tumor marker, including numbers of missing values. | Results, appendix |  |
| *Analysis and presentation* | | | |
| 14 | Show the relation of the marker to standard prognostic variables. | Tables, appendix |  |
| 15 | Present univariable analyses showing the relation between the marker and outcome, with the estimated effect (e.g., hazard ratio and survival probability). Preferably provide similar analyses for all other variables being analyzed. For the effect of a tumor marker on a time-to-event outcome, a Kaplan-Meier plot is recommended. | Results, figures, appendix |  |
| 16 | For key multivariable analyses, report estimated effects (e.g., hazard ratio) with confidence intervals for the marker and, at least for the final model, all other variables in the model. | Tables, appendix |  |
| 17 | Among reported results, provide estimated effects with confidence intervals from an analysis in which the marker and standard prognostic variables are included, regardless of their statistical significance. | Tables, appendix | Included standard prognostic variables |
| 18 | If done, report results of further investigations, such as checking assumptions, sensitivity analyses, and internal validation. | Yes | Internally validated in a new cohort, then independently validated in an external cohort, will further validated in prospective cohort (NCT03928652). |
| **DISCUSSION** | | | |
| 19 | Interpret the results in the context of the pre-specified hypotheses and other relevant studies; include a discussion of limitations of the study. | Discussion |  |
| 20 | Discuss implications for future research and clinical value. | Discussion |  |

**Supplementary Table 2. Clinicopathological features of the 60 colorectal cancer patients from whom with and without relapse (rCRC and nrCRC) operation specimens with adjuvant chemotherapy were collected**

| Characteristics | rCRC (n=30) | nrCRC (n=30) | P value |
| --- | --- | --- | --- |
| Age |  |  | 0.778 |
| ≥65 | 20 (66.7) | 22 (73.3) |  |
| <65 | 10 (33.3) | 8 (26.7) |  |
| Sex |  |  | 1.0 |
| Male | 11 (36.7) | 11 (36.7) |  |
| Female | 19 (63.3) | 19 (63.3) |  |
| T stage |  |  | 1.0 |
| T2 | 2 (6.7) | 2 (6.7) |  |
| T3 | 28 (93.3) | 28 (93.3) |  |
| N stage |  |  |  |
| N0 | 10 (33.3) | 13 (43.3) | 0.363 |
| N1 | 13 (43.3) | 14 (46.7) |  |
| N2 | 7 (23.3) | 3 (10.0) |  |
| TNM stage |  |  | 0.595 |
| II | 10 (33.3) | 13 (43.3) |  |
| III | 20 (66.7) | 17 (56.7) |  |
| Curative resection | 30 (100) | 30 (100) | 1.0 |
| Adjuvant chemotherapy | 30 (100) | 30 (100) | 1.0 |

Data are n (%).

**Supplementary Table 3. The association of disease-free survival with 4 markers from multiplex immunohistochemistry in training cohort**

| characteristics | HR (95% CI) | P value |
| --- | --- | --- |
| Hscore of FHL3 in tumor area | 1.003 (1 - 1.007) | 0.06 |
| Hscore of TGFBI in stromal area | 1.005 (1.001 - 1.008) | 0.005 |
| Hscore of GGA1 tumor area | 0.992 (0.983 - 1.001) | 0.08 |
| Hscore of NDUFS7 tumor area | 1.002 (0.998 - 1.006) | 0.4 |

**Supplementary Table 4. Coefficients for the final prediction algorithm**

| Variables | coefficeints | Exp (coef) | Se (coef) | z | p |
| --- | --- | --- | --- | --- | --- |
| Hscore of TGFBI in stromal area | 0.004 | 1.004 | 0.002 | 2.462 | 0.01 |
| Hscore of FHL3 in tumor area | 0.003 | 1.003 | 0.002 | 1.397 | 0.16 |
| Hscore of GGA1 tumor area | -0.006 | 0.994 | 0.005 | -1.392 | 0.16 |

**Supplementary Table 5. Number of events for the different groups**

| Number of events | Training cohort (n=203) | | Internal validation cohort (n=204) | | External validation cohort (n=333) | |
| --- | --- | --- | --- | --- | --- | --- |
|  | Low PS (n=77) | High PS  (n=126) | Low PS  (n=63) | High PS (n=141) | Low PS (n=104) | High PS (n=229) |
| Relapse | 12 | 53 | 8 | 48 | 15 | 83 |
| Death | 10 | 44 | 8 | 36 | 13 | 72 |

**Supplementary Table 6. Five-year disease-free survival and overall survival estimates for the different groups**

| 5-year survival | Training cohort  (n=203) | | Internal validation cohort (n=204) | | External validation cohort (n=333) | |
| --- | --- | --- | --- | --- | --- | --- |
|  | Low PS  (n=77) | High PS  (n=126) | Low PS  (n=63) | High PS  (n=141) | Low PS  (n=104) | High PS  (n=229) |
| DFS (95% CI) | 85.7  (78.2-93.9) | 64.3  (56.4-73.2) | 88.9  (81.5-97.0) | 71.5  (64.4-79.4) | 86.6  (80.1-93.7) | 66.9  (60.9-73.6) |
| OS (95% CI) | 89.6  (83.0-96.7) | 69.8  (62.3-78.3) | 90.5  (83.5-98.0) | 79.3  (72.8-86.3) | 89.6  (83.7-95.9) | 70.5  (64.6-77.0) |

DFS, disease-free survival; OS, overall survival; PS, proteomic signature

**Supplementary Table 7. Multivariable overall survival analyses in each cohort**

| Variables | HR | 95% CI | P value |
| --- | --- | --- | --- |
| Training cohort (n=203) |  |  |  |
| Age (≥65 vs. <65) | 2.46 | 1.35-4.47 | 0.003 |
| Tumor location (rectum vs. colon) | 1.80 | 1.04-3.12 | 0.036 |
| pN stage (N1-2 vs. N0) | 1.81 | 1.004-3.26 | 0.048 |
| PS (high vs. low) | 2.53 | 1.26-5.10 | 0.009 |
| Internal validation cohort (n=204) |  |  |  |
| pT stage (T4 vs. T1-3) | 3.04 | 1.52-6.08 | 0.002 |
| pN stage (N1-2 vs. N0) | 2.87 | 1.46-5.66 | 0.002 |
| PS (high vs. low) | 1.98 | 0.92-4.28 | 0.082 |
| External validation cohort (n=333) |  |  |  |
| pT stage (T4 vs. T1-3) | 2.48 | 1.47-4.19 | <0.001 |
| N stage (N2 vs. N1) | 2.78 | 1.72-4.48 | <0.001 |
| CEA (≥5 vs. <5 ng/L) | 2.53 | 1.61-3.96 | <0.001 |
| PS (high vs. low) | 2.93 | 1.58-5.42 | <0.001 |

We calculated hazard ratios and p values using an adjusted multivariate Cox proportional hazards regression model, including proteomic signature (high risk vs. low risk), sex (male vs. female), age (≥ 60 years vs. 60 years), N stage (N1-2 vs. N0; N2 vs. N1), T stage (T4 vs. T1-3), MMR, (dMMR vs. pMMR) histology (low vs. high), location (rectum vs. colon), lymph nodes examined (≥12 vs. <12). and CEA (≥5 vs. <5 ng/L). OS, overall survival; PS, proteomic signature; CEA, carcinoembryonic antigen; HR, hazard ratio; CI, confidence interval. We selected variables with the backward stepwise approach, the p value threshold was 0·05 (p> 0·05) for removing insignificant variables from the model. Only variables that were significantly associated with survival are presented.

**Supplementary Table 8. The C index of four risk factors in each cohort**

|  | Training cohort | | Internal validation cohort | | External validation cohort | |
| --- | --- | --- | --- | --- | --- | --- |
| Factors | C-index | 95% CI | C-index | 95% CI | C-index | 95% CI |
| PS | 0.62 | 0.56-0.67 | 0.59 | 0.54-0.65 | 0.59 | 0.55-0.63 |
| N stage | 0.60 | 0.54-0.67 | 0.59 | 0.53-0.65 | 0.62 | 0.58-0.65 |
| Tumor location | 0.57 | 0.50-0.64 | 0.57 | 0.50-0.63 | 0.51 | 0.46-0.56 |
| Age | 0.63 | 0.56-0.69 | 0.56 | 0.49-0.62 | 0.54 | 0.50-0.59 |

PS, proteomic signature; HR, hazard ratio; CI, confidence interval.

**Supplementary Figure 1. Experimental workflow**

(a) Experimental workflow for generating MS-based proteomic data; (b) Workflow of multiplex immunohistochemistry staining and image analysis. LC-MS/MS=liquid chromatography tandem mass spectrometry.

**Supplementary Figure 2. Longitudinal quality control of mass spectrometry using tryptic digest of HEK293T cells**


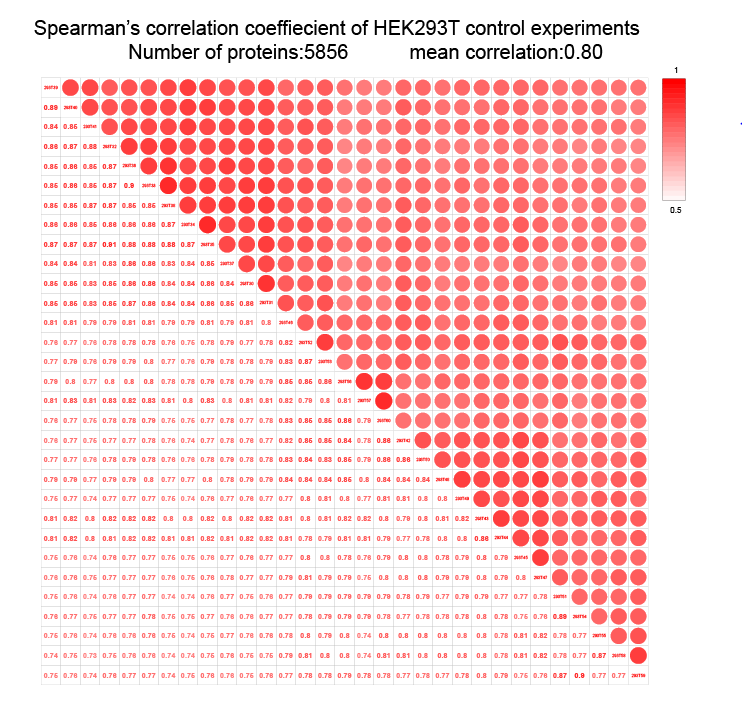


**Supplementary Figure 3. Multiplex immunohistochemistry staining of three proteins in formalin-fixed, paraffin-embedded tissues.**


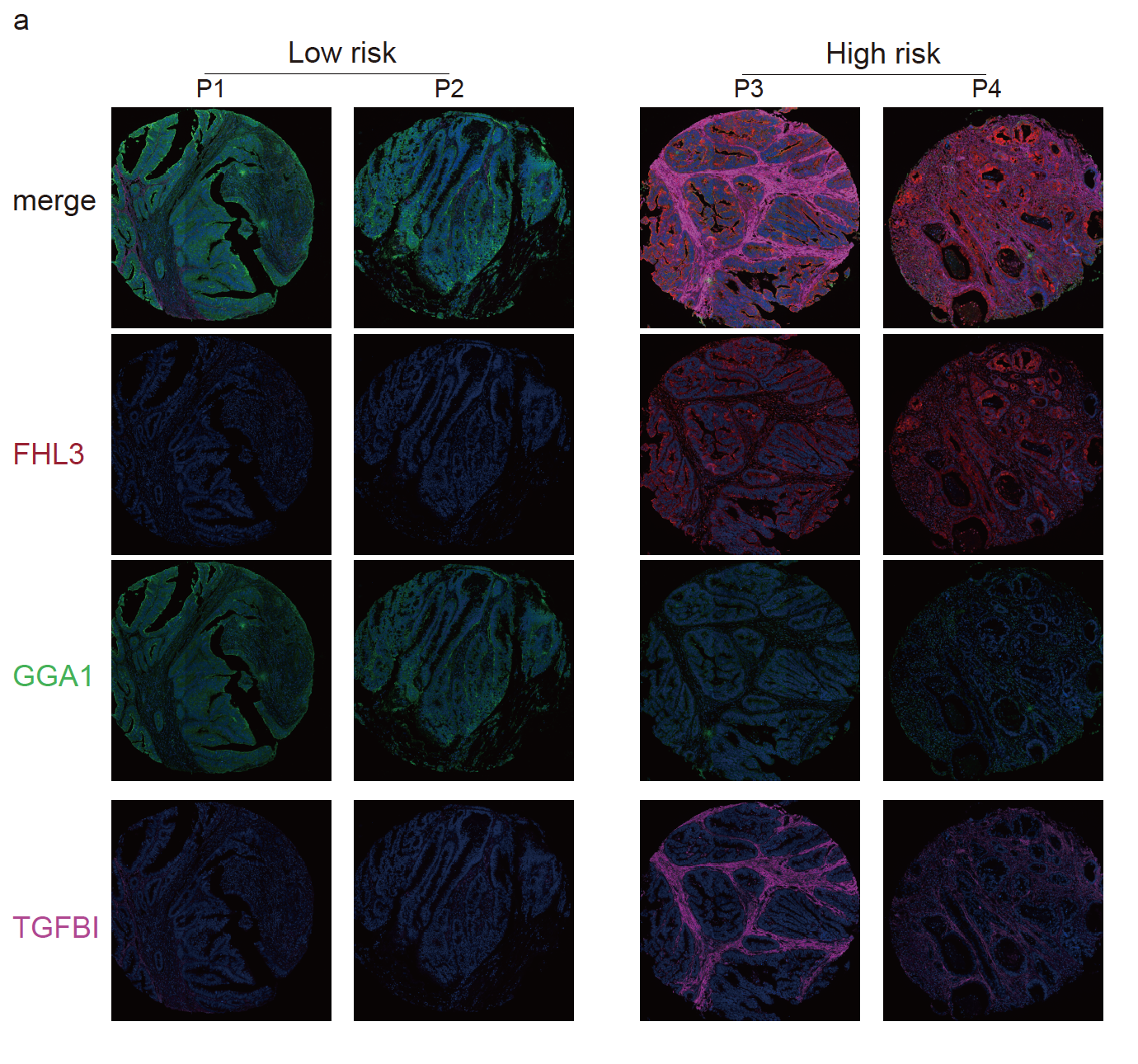


Representative of multiplex immunohistochemistry staining of three proteins in formalin-fixed, paraffin-embedded tissues of patients with low risk or high risk.

**Supplementary Figure 4. Antibody validation for four proteins**

**
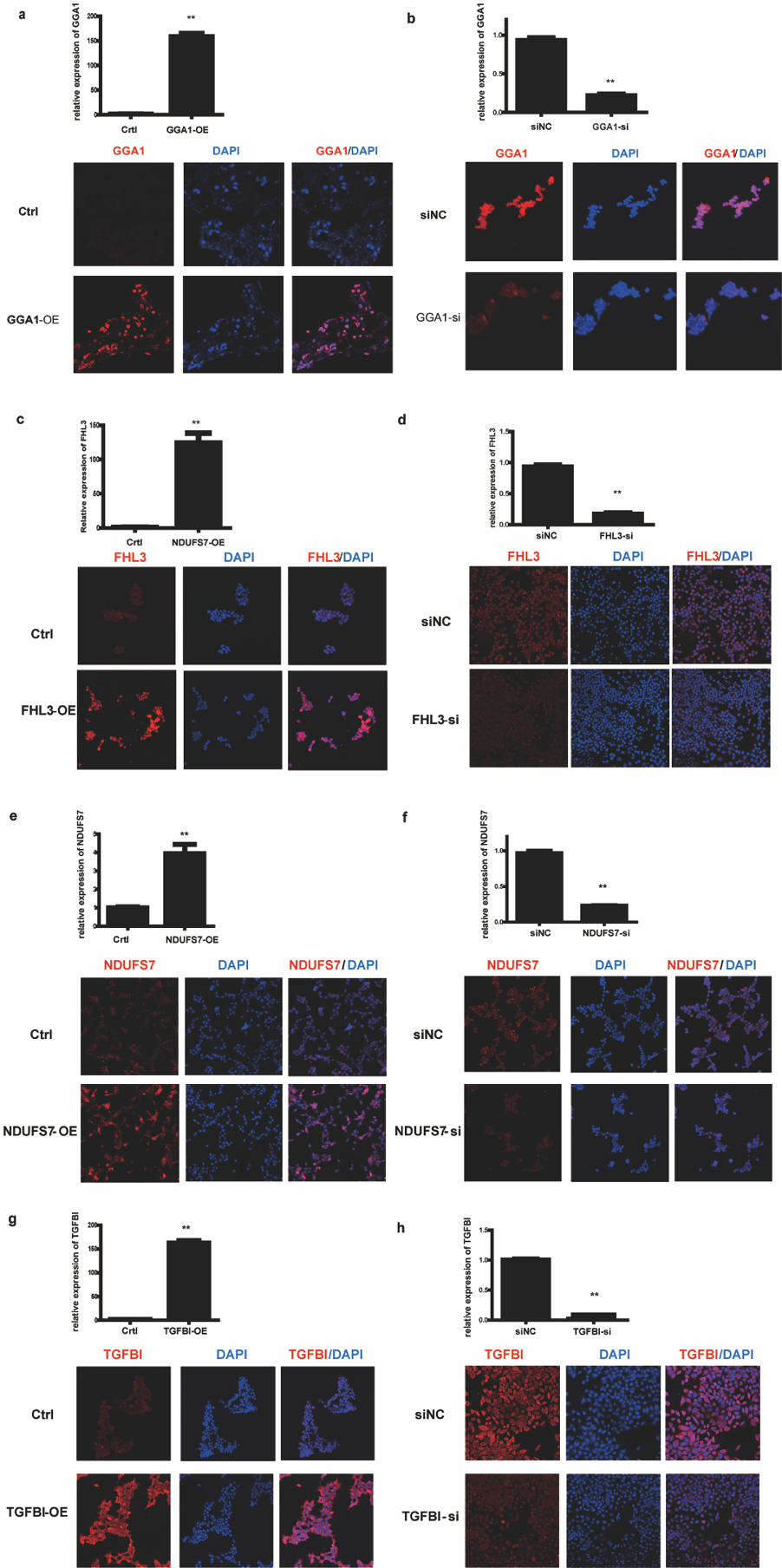
**

The specificities of antibodies employed were validated by siRNA knockdown or recombinant expression in immunofluorescence. A-B. GGA1; C-D. FHL-3; E-F. NDUFS7; G-H. TGFBI

**Supplementary Figure 5. Marker selection**

Two steps were used for feature selection. (a) Wilcox test was used to analysis differentially expressed proteins between tumor and non-tumor, relapse group and non-relapse group respectively. differentially expressed proteins define as significantly over expressed in both tumor and relapse group or significantly down expressed in both tumor and relapse group. (b) LASSO logistic algorithm and SVM logistic model to were used to select 4 out of 12 differentially expressed proteins. (c) Expression level of FHL3, GGA1, NDUFS7, TGFBI in stromal and tumor cells, Line represents the median value, box spans 25th to 75th percentile, and whiskers span 5th to 95th percentile. P value determined by student’s t-test, **** represents p<0.0001.

**Supplementary Figure 6.** **GGA1, FHL3, TGFBI, and NDUFS7 associated with disease-free survival of patients receiving adjuvant chemotherapy**


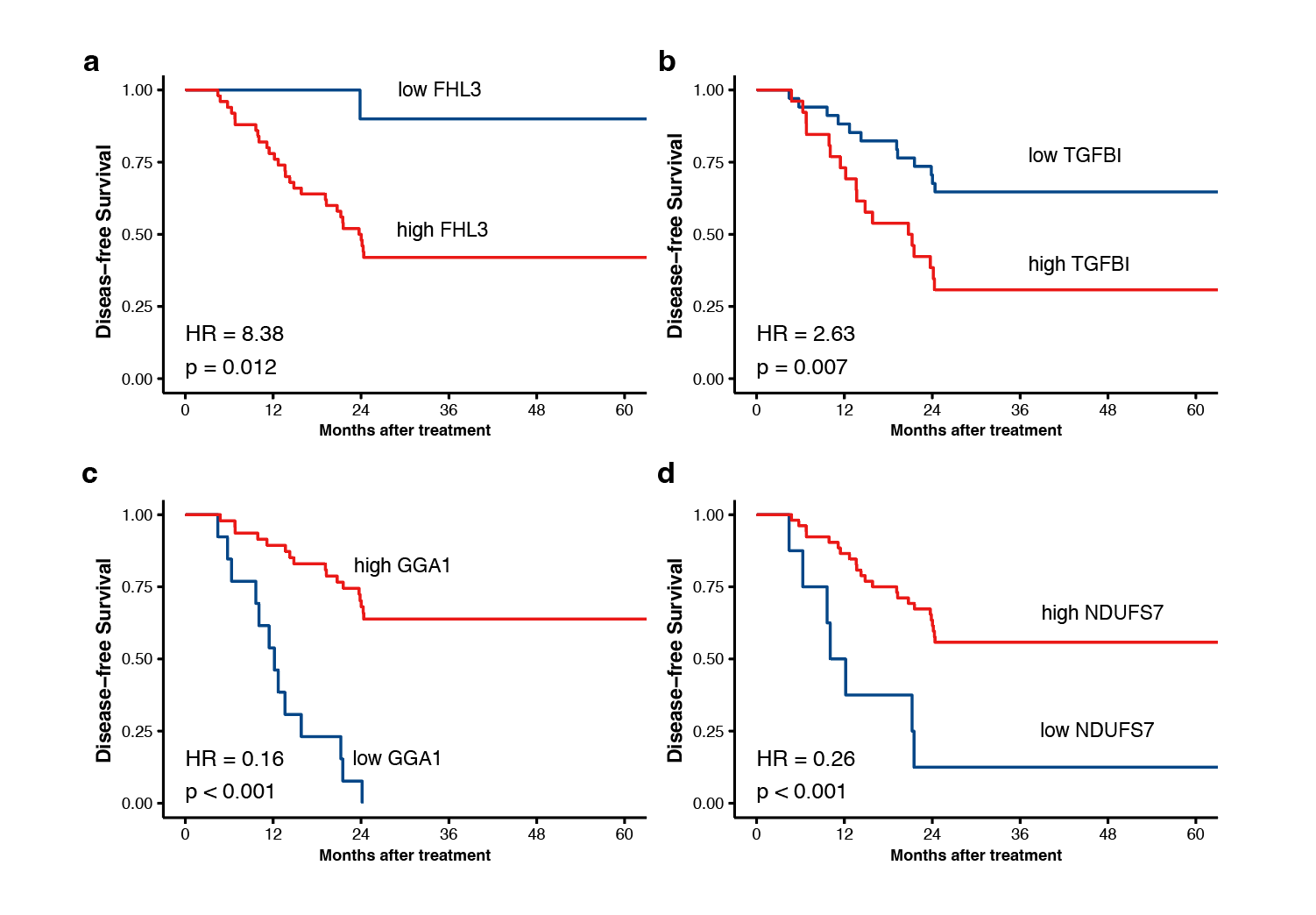


ACT=adjuvant chemotherapy, HR=hazard ratio

**Supplementary Figure 7. X-tile plots of risk value in the training set.**


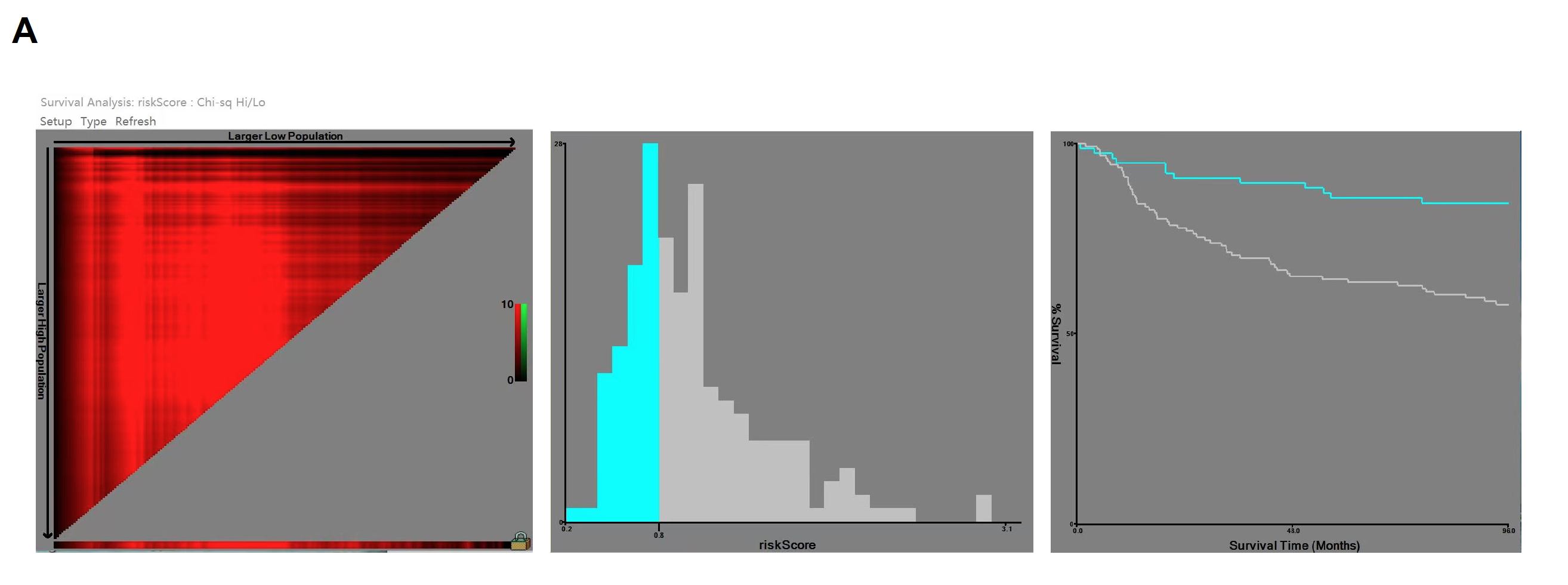


Coloration of the plot represents the strength of the association at each division, ranging from low (dark, black) to high (bright, red or green). Red represents inverse association between marker expression and survival, whereas green represents direct association.

**Supplementary Figure 8. ROC curve comparing the sensitivity and specificity of the nomogram**


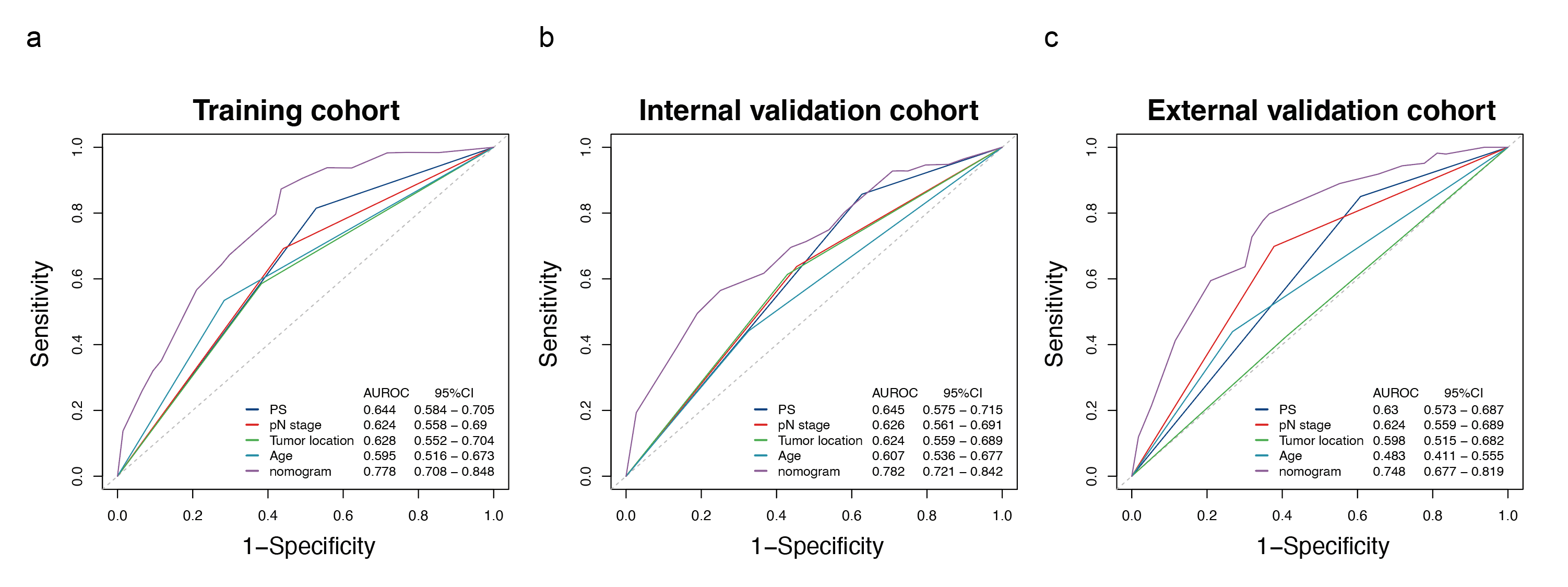


(a) the training cohort, (b) the internal validation cohort, (c) the external validation cohort; AUC, area under the curve; CI, confidence interval.

**Supplementary Figure 9. Kaplan-Meier curves for OS according to the PS.**

**
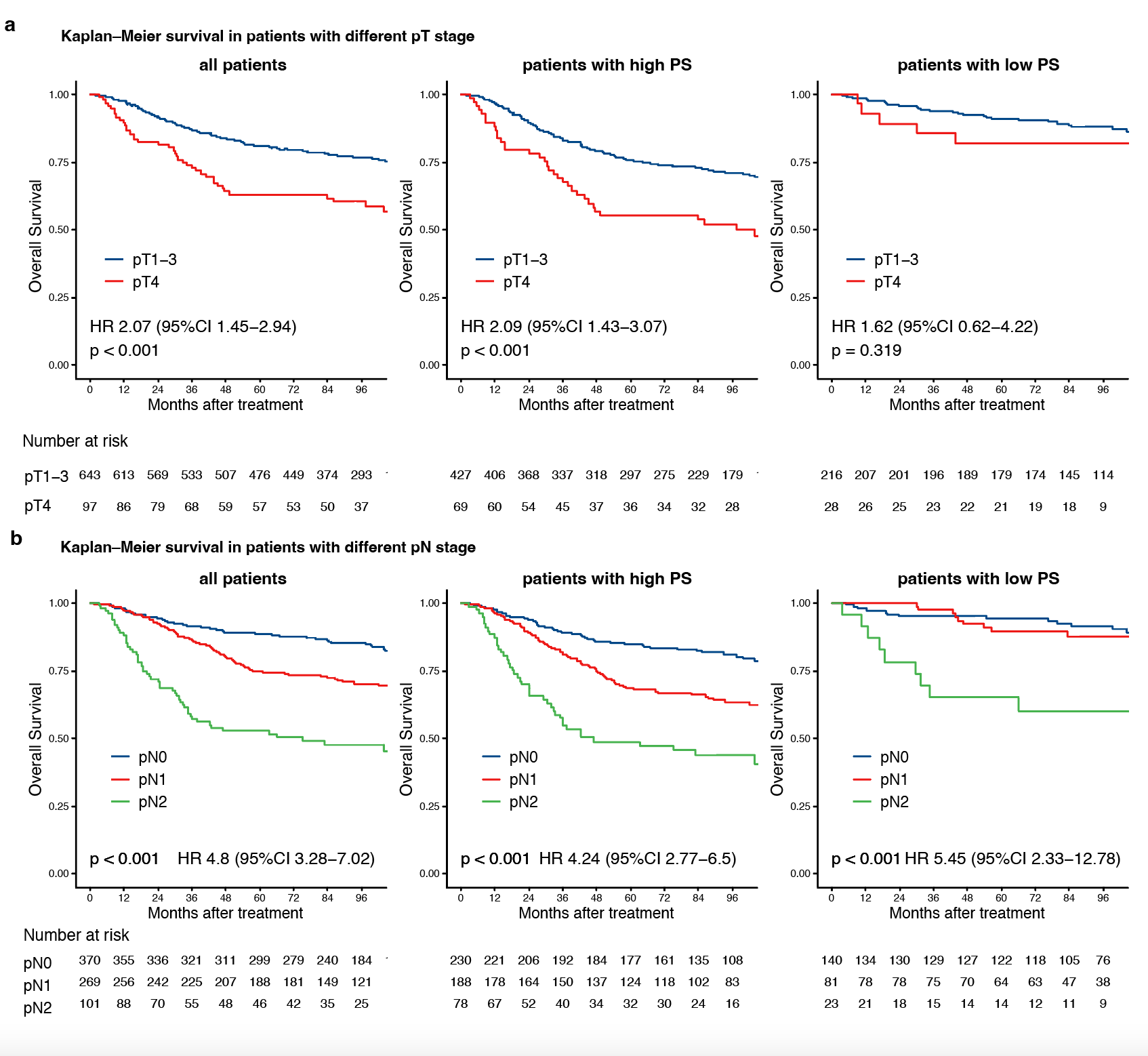
**

The results are shown for all patients (n =740, left), patients with a high PS (n = 496, middle), and patients with a low PS (n = 244, right). The results are also stratified according to pT stage (a), and pN stage (b). p values were calculated using two-sided log-rank test. PS=proteomic signature, OS=overall survival, HR=hazard ratio, CI= confidential interval.

**Supplementary Figure 10. ACT benefits based on OS according to pN stage and PS.**

**
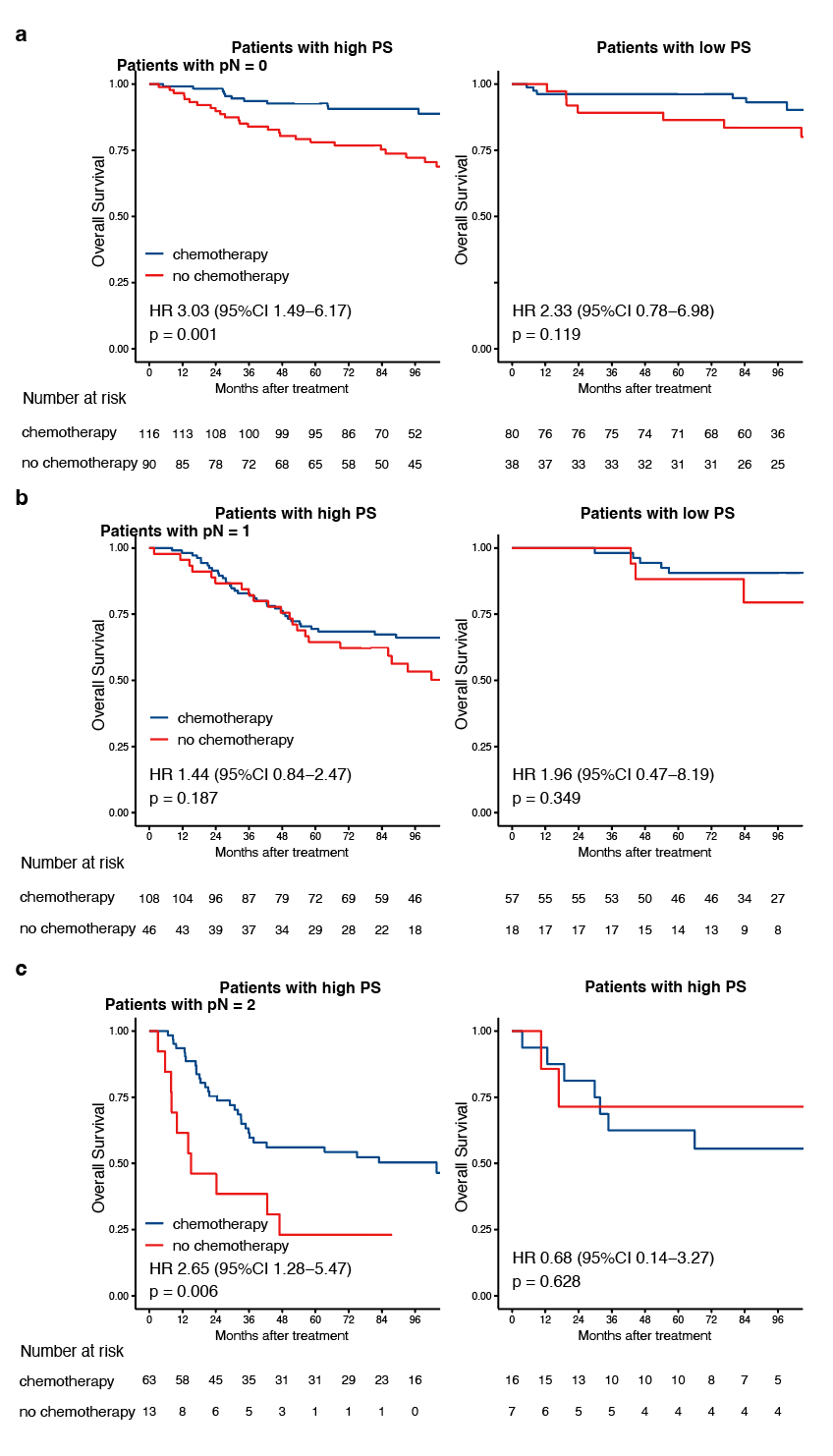
**

a–c Kaplan-Meier OS curves are shown for patients according to their use of ACT. In addition, patients with a high RS (left) were stratified according to pN0 (n = 206, upper), pN1 (n = 154, middle), and pN2 (n = 76, bottom). Patients with a low RS (right) were also stratified according to pN0 (n = 118, upper), pN1 (n = 75, middle), and pN2 (n = 23, bottom). p values were calculated using two-sided log-rank test. PS=proteomic signature, OS=overall survival, HR=hazard ratio, CI= confidential interval.
