# Supplementary material for: High-throughput proteomics profiling-derived signature associated with chemotherapy response and survival for stage II/III colorectal cancer
Source: NPJ Precis Oncol. 2023 May 31;7:50. doi: 10.1038/s41698-023-00400-0 (PMC10232411; doi:10.1038/s41698-023-00400-0)
Supplement: Supplementary file 2 — REPORTING SUMMARY [file 41698_2023_400_MOESM2_ESM.pdf]

nature portfolio

Corresponding author(s): Xiao-Jian Wu, Jun Qin, Zi-Huan Yang and Ping Lan

Last updated by author(s): May 8, 2023

Reporting Summary

Nature Portfolio wishes to improve the reproducibility of the work that we publish. This form provides structure for consistency and transparency in reporting. For further information on Nature Portfolio policies, see our Editorial Policies and the Editorial Policy Checklist.

Statistics

For all statistical analyses, confirm that the following items are present in the figure legend, table legend, main text, or Methods section.

n/a	Confirmed	
☐	☒	The exact sample size (*n*) for each experimental group/condition, given as a discrete number and unit of measurement
☐	☒	A statement on whether measurements were taken from distinct samples or whether the same sample was measured repeatedly
☐	☒	The statistical test(s) used AND whether they are one- or two-sided<br>*Only common tests should be described solely by name; describe more complex techniques in the Methods section.*
☐	☒	A description of all covariates tested
☐	☒	A description of any assumptions or corrections, such as tests of normality and adjustment for multiple comparisons
☐	☒	A full description of the statistical parameters including central tendency (e.g. means) or other basic estimates (e.g. regression coefficient) AND variation (e.g. standard deviation) or associated estimates of uncertainty (e.g. confidence intervals)
☐	☒	For null hypothesis testing, the test statistic (e.g. *F*, *t*, *r*) with confidence intervals, effect sizes, degrees of freedom and *P* value noted<br>*Give P values as exact values whenever suitable.*
☒	☐	For Bayesian analysis, information on the choice of priors and Markov chain Monte Carlo settings
☒	☐	For hierarchical and complex designs, identification of the appropriate level for tests and full reporting of outcomes
☒	☐	Estimates of effect sizes (e.g. Cohen's *d*, Pearson's *r*), indicating how they were calculated

Our web collection on statistics for biologists contains articles on many of the points above.

Software and code

Policy information about availability of computer code

Data collection	No software was used.
Data analysis	All statistical analyses were performed by R software (version 4.1.0;R Foundation for Statistical Computing, Vienna, Austria). Least Absolute Shrinkage and Selection Operator (LASSO) and support vector machine-recursive feature elimination (SVM-RFE) analyses were done using the “glmnet” package(v4.1) and e1071(v1.7). Nomograms and calibration plots were generated using rms package(v6.3). GSEA analysis was generated using “clusterprofiler” package(v4.0.5).

For manuscripts utilizing custom algorithms or software that are central to the research but not yet described in published literature, software must be made available to editors and reviewers. We strongly encourage code deposition in a community repository (e.g. GitHub). See the Nature Portfolio guidelines for submitting code & software for further information.

Data

Policy information about availability of data

All manuscripts must include a data availability statement. This statement should provide the following information, where applicable:

- Accession codes, unique identifiers, or web links for publicly available datasets
- A description of any restrictions on data availability
- For clinical datasets or third party data, please ensure that the statement adheres to our policy

Deidentified clinical data can be made available with publication through the corresponding author after approval of a proposal with a signed data acces agreement.

Only deidentified data that underlie results reported in this Article can be shared with investigators who submit an approved proposal. After approval of a proposal, clinical data can be shared through a secure online platform after signing a data access agreement. The proteomic data has been deposited on iProX (https://www.proteomexchange.org/, accession number:IPX0003266000). iProX is an official member of ProteomeXchange Consortium which includes PRIDE, PeptideAtlas, MassIVE, jPOST, iProx, and Panorama Public. The Consortium was established to provide globally coordinated standard data submission and dissemination pipelines involving the main proteomics repositories, and to encourage open data policies in the field

Research involving human participants, their data, or biological material

Policy information about studies with human participants or human data. See also policy information about sex, gender (identity/presentation), and sexual orientation and race, ethnicity and racism.

Reporting on sex and gender	Both male and female were included in the study, Detailed information is included in Supplementary Table 2
Reporting on race, ethnicity, or other socially relevant groupings	N/A
Population characteristics	all clinical characteristic are described in Supplementary Table 2.
Recruitment	We excluded samples if the patient met the exclusion criteria (clinical quality control, eg, metastatic cancer, received previous treatment with any anticancer therapy, stage I disease, or missing mortality or recurrence data). All patients with stage II/III CRC received curative-intent surgery, and no patients received preoperative antitumor treatment. After radical surgery, a proportion of patients received available standard systemic treatment, include fluorouracil (FU) or capecitabine with or without oxaliplatin.
Ethics oversight	This study was approved by the Institutional Review Board under 2020ZSLYEC-229.

Note that full information on the approval of the study protocol must also be provided in the manuscript.

Field-specific reporting

Please select the one below that is the best fit for your research. If you are not sure, read the appropriate sections before making your selection.

☒ Life sciences ☐ Behavioural & social sciences ☐ Ecological, evolutionary & environmental sciences

For a reference copy of the document with all sections, see nature.com/documents/nr-reporting-summary-flat.pdf

Life sciences study design

All studies must disclose on these points even when the disclosure is negative.

Sample size	We included 740 samples which passed quality control for the final analysis
Data exclusions	We excluded samples if the patient met the exclusion criteria (clinical quality control, eg, metastatic cancer, received previous treatment with any anticancer therapy, stage I disease, or missing mortality or recurrence data). All patients with stage II/III CRC received curative-intent surgery, and no patients received preoperative antitumor treatment. After radical surgery, a proportion of patients received available standard systemic treatment, include fluorouracil (FU) or capecitabine with or without oxaliplatin.
Replication	N/A
Randomization	N/A
Blinding	N/A

Reporting for specific materials, systems and methods

We require information from authors about some types of materials, experimental systems and methods used in many studies. Here, indicate whether each material, system or method listed is relevant to your study. If you are not sure if a list item applies to your research, read the appropriate section before selecting a response.

Materials & experimental systems

n/a	Involved in the study	
☐	☒	Antibodies
☐	☒	Eukaryotic cell lines
☒	☐	Palaeontology and archaeology
☒	☐	Animals and other organisms
☒	☐	Clinical data
☒	☐	Dual use research of concern
☒	☐	Plants

Methods

n/a	Involved in the study	
☒	☐	ChIP-seq
☒	☐	Flow cytometry
☒	☐	MRI-based neuroimaging

Antibodies

Antibodies used	GGA1 (H00026088-M01, NOVUS, USA), FHL3 (11028-2-AP, Proteintech, China), NDUFS7 (15728-1-AP, Proteintech, China) and TGFBI (ab170874, Abcam, USA) ,MLH1 (clone ES05; Zhong Shan Jin Qiao, Beijing, China), MSH2 (clone RED2; Zhong Shan Jin Qiao, Beijing, China), MSH6 (clone UMAB258; Zhong Shan Jin Qiao, Beijing, China) and PMS2 (clone EP51; Zhong Shan Jin Qiao, Beijing, China).
Validation	antibodies were used as described in the manuscript, are commercially available.

Eukaryotic cell lines

Policy information about cell lines and Sex and Gender in Research

Cell line source(s)	All cell line were obtained from ATCC.
Authentication	All cell line were certified by the indicated cell bank and routinely authenticated by morphologic inspection.
Mycoplasma contamination	all cell line were tested to confirm they were free for Mycoplasma.
Commonly misidentified lines (See ICLAC register)	Commonly misidentified lines were not used.
